# A Guide to the Clinical Examination of the Blood, for Diagnostic Purposes

**Published:** 1899-11

**Authors:** 


A GUIDE TO THE CLINICAL EXAMINATION OF THE BLOOD, FOR DIAGNOSTIC PURPOSES. By Richard C. Cabot, M. D., Boston. With colored plates and engravings. Third revised edition; 440 pages. Price, $3.25. New York: William Wood & Co., 1899.

This edition of Prof. Cabot's book has been thoroughly revised. The many useful and practical discoveries that have been made since the second edition came out have made it necessary to rewrite much of the work, and to leave out much that was out of date. The methods of examination and study are clinical, and the technique is given with such care that any physician may learn to make reliable blood examinations for himself. There are so many diseases where a knowledge of the blood, as understood now, will aid in the treatment that no physician should allow himself to neglect this important subject. It requires very little study, if you have a microscope and know how to use it, to be able to make a blood-count and to find the plasmodium malaria, and it requires only a few minutes, by the use of a hœmoglobmometer, to determine the per centage of hæmoglobin.

The principal additions to this well and favorably known book include an account of Oliver's tintometer and hæmoglogmometer, and new chapters on primary anæmias and leukæmia, Muller's "blood-dust," a new constituent of blood, new observations on poisoning, on aneurisms, on cretinism, etc., have been added. This book should be in the library of every good physician, and every good physician should give his patients the benefit of the information contained therein. Much of the subject matter of this book is comparatively new. B.

